# Comparing carbon footprints of sheep farming systems in semi-arid regions of India: A life cycle assessment study

**DOI:** 10.1371/journal.pone.0292066

**Published:** 2024-01-30

**Authors:** Srobana Sarkar, B. Lal, Priyanka Gautam, R. S. Bhatt, A. Sahoo

**Affiliations:** 1 ICAR-Central Sheep and Wool Research Institute, Malpura, Rajasthan, India; 2 ICAR-Indian Institute of Pulses Research, Regional Research Centre, Bikaner, Rajasthan, India; 3 ICAR-National Research Centre on Camel, Bikaner, Rajasthan, India; ICAR-Indian Institute of Soil Science, INDIA

## Abstract

Carbon foot prints (CFs) studies based on life cycle assessment between sheep farming systems and green house gases (GHG) emissions is one of the best indicators to quantify the amount of GHG emissions per kg of product. Therefore, a life cycle assessment (LCA) study was conducted for three different sheep farming systems i.e. intensive system (stall fed only), semi-intensive (grazing with supplementation) and extensive system (grazing only) under semiarid region of India to assess the carbon cost of sheep rearing. The total CFs were estimated to be 16.9, 15.8 and 17.1 kg CO_2_-eq in intensive, semi-intensive and extensive system of grazing indicating semi-intensive system to be most carbon (C) efficient. For 1kg mutton production in semi-intensive and intensive system, around 30% and 24% CFs were contributed from enteric fermentation and feed respectively, whereas, in extensive system, the contribution of enteric fermentation increased up to 50%. The carbon foot prints analysis gives an insight of carbon inputs used but the amount of CO_2_ sequestered in soil making LCA a holistic approach for estimating GHG emissions from livestock.

## Introduction

Sheep farming has always remained an integral part of global agricultural wealth because of their multifaceted role in meat, milk and wool production. Sheep can conveniently adapt to any production system, ranging from extensive farming to organised intensive rearing systems along with wide climatic variation. In spite of their small contribution in total milk and meat production of the world, yet sheep farming plays a significant role in the economy of developing countries. Sheep sector alone produces around 40% and 62% of the total milk and meat from small ruminants, respectively [1]. As per 20^th^ Livestock census of India [2] the estimated sheep population is 74.26 million in the country with an increase of 14.1% over the previous census thereby depicting the demand of sheep enterprise among marginal farmers. Therefore, both breeding and nutritional interventions are required to enhance the total and per unit production from this sector meet out the demands of food and fibre for upsurging human population.

For communicating the impacts of climate change to food producers, researchers and policy makers, determination of carbon footprint is becoming very popular [3]. The application of LCA in livestock production systems has emerged to be a new research venture for estimating carbon cost of the production system [4]. The LCA studies relevant to small ruminant rearing system and their implications on environment have been conducted in European countries however; scanty scientific literatures are available on CFs of sheep production in different grazing systems under Indian scenario. Researches on carbon footprints (CFs) associated to sheep farming were mainly concentrated in European countries and reports suggested around 254 and 67.1 Mt CO_2_-eq GHG emissions are contributed by sheep production and sheep milk system, respectively [1, 5]. Therefore, the present study was conducted to determine the CFs of 1 kg mutton production from sheep reared under intensive (stall fed only), semi-intensive (grazing + stall fed) and extensive (grazing on pasture land only) systems. The study was planned with the following objectives: 1) to estimate the carbon footprints of sheep farming under different grazing systems, and 2) to compare the efficiency and sustainability of grazing systems.

## Materials and methods

### Sheep farming under different grazing systems

In the present study, data from around 100 hectares of farm with 500 hundred sheep (Malpura and Avishaan) reared under different rearing systems was considered in the study. The farm area for sheep rearing was developed for more than 20 years by ICAR-CSWRI, Avikanagar. Key characteristics of the different rearing systems are given in Table 1. Average annual meat yield per sheep varied from 16 kg in extensive system, 19 kg in intensive and >20 in semi-intensive system.

**Table 1:** Technical description of the studied sheep farms in semi-arid region of India.

	Intensive system	Semi-intensive system	Extensive system
**Farm characteristics**			
Area (ha)	25.6	42.5	34.4
No. of sheep	150	240	120
LSU/ha	8	6	3.5
Dry matter intake (kg/d/sheep)	1.04	1.19	0.98
Fodder intake (g/day/sheep)	638.48	774.87	988.00
Concentrate (g/day/sheep)	411.46	237.96	0.00
Digestibility of dry matter (%)	61.39	58.47	49.72
Grazing hours/d	0	6	8
Average daily gain (g/day)	126.96	146.81	64.80
CH_4_ (g/d/sheep)	20.25	23.21	25.58
CH_4_ (g/kg DMI)	19.49	21.97	27.56
CH_4_ (g/kg DDMI)	35.59	38.63	54.73
**Input**			
Concentrate (kg/sheep/year)	150.18	86.85	0
Fodder (kg/sheep/year)	233.045	282.83	360.62
Oil (litres/year)	45	35	15
Electricity (kWh/year)	24528.0	14016	3504
Fertilizer (N, P and K) kg/ha	290	220	0
**Output**			
Carcass yield (kg/sheep)	19.69	20.79	16.10
Wool (kg/sheep/year)	1.30	1.38	1.17
Milk (lit/ewe/year)	60.72	52.24	37.45

LSU; livestock unit, CH_4;_ methane, DMI; dry matter intake, DDMI; digestible dry matter intake, N; nitrogen, P; phosphorus, K; potassium

### Feeding systems

#### i) Intensive system (stall fed only)

Initially range management with feed supplements is the sheep production system was followed in the country, however, with the changing scenario of shrinking grazing lands, intensive feeding is widely adapted for mutton production. Generally, diets having 40:60 or 70:30 roughage and concentrate ratio are used for mutton production commercially. But to harvest better gain under intensive feeding, cafeteria system of feeding management was applied in our farm. Wherein, the concentrate and roughage components were offered in separate containers and the animals were given free choice to adjust its roughage/concentrate ratio.

#### ii) Semi-intensive system (grazing+ stall fed)

Under this system, sheep had access to adequate quantity and quality of vegetation from July-October. Apart from that, the growth cycle of vegetation in arid and semi-arid environment is completed in less than 90 and in between 90–180 days, respectively; therefore, animals were bound to survive on sparse vegetation for most part of the year. Free grazing for 6 hrs with concentrates supplementation @ 150–200 g/ sheep/ day was adopted under this system.

#### iii) Extensive system (grazing on natural pasture vegetation)

Under the extensive system, the animals were sent to grazing for a period of 8 hrs without any extra concentrate supplementation. The grazing area consisted of shrubs and trees besides grasses, either in isolation or in large and small patches with variable density. The different combinations of grass species present in the pasture were *Sehima-Dicanthium* type, *Dicanthium-Cenchrus-Lasiurus* type, *Phragmites-Sachharum-Imperate* type. Bushes of *Zizyphus numularia* also constitute sizeable proportion of grass cover.

### Life cycle assessment and inventory analysis

**Fig 1:**
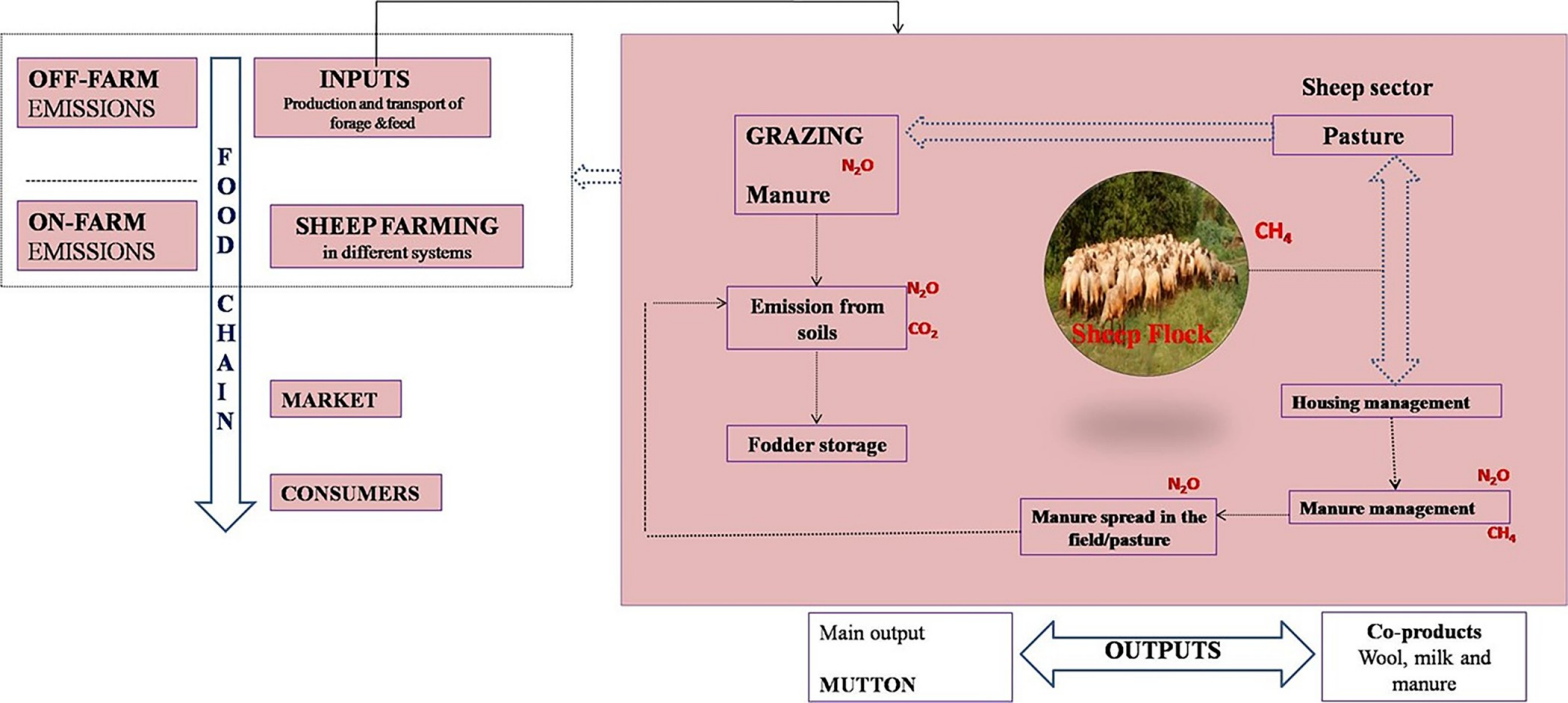


Carbon footprint values were calculated using the life cycle assessment (LCA) methodology (Fig 1).

The factors characterized for global warming potential (GWP) of greenhouse gases were 25 and 265 for CH_4_ and N_2_O, respectively based on the IPCC standards. The functional unit considered is 1 kg of sheep output (meat). The dry biomass produced in the farm was used as a feed for the sheep in the sector and the main functional unit is the kg of carcass weight sold. The system boundaries were from cradle to farm gate included agricultural inputs, field agricultural processes for forage cultivation, direct and indirect emissions of CH_4_, N_2_O and CO_2_ from sheep farm, direct emissions are those, which occur on farm, and indirect emissions can be attributed in the farm but occurs somewhere else. According to the system boundary, all GHG emissions that take place on the farm are shown in Tables 2 and 3 and the equations and emissions factors that have been used. Most of them correspond to the IPCC guidelines [6]. The resources consumed and emission factors associated to each process involved in fodder production and sheep farming were quantified in the inventory analysis. Data from previously followed grazing systems in farm over the years were used to calculate carbon footprints.

**Table 2:** Emission factors used in the sheep farms for quantification of off-farm emissions.

Item	Emission factors	Reference
Mutton	23.8CO_2_eq/kg mutton	FAO, 2013
N	5.27 kg CO_2_eq/kg N	Gac et al., 2010 [20]
P	0.566 kg CO_2_eq/kg P_2_O_5_	Gac et al., 2010 [20]
K	0.444 kg CO_2_eq/kg K_2_O	Gac et al., 2010 [20]
Herbicide	8.98 kg CO_2_eq/kg active material	Gac et al., 2010 [20]
Electricity	0.29 kg CO_2_eq/kWh	Gac et al., 2010 [20]
Diesel	2.664 kg CO_2_eq/litre	IPCC, 2006

CO_2eq;_ carbon dioxide equivalent, P_2_O_5_; Phosphorus pentoxide, N; nitrogen, P; phosphorus, K; potassium, K_2_O; potassium oxide.

**Table 3:** Emission factor used in the baseline scenario of the sheep for quantification of on-farms GHG emissions.

Source of GHG emission	Pollutant	EF	Unit	Reference
Enteric fermentation	CH_4_	3.61 kg/head/year	kg CH_4_/year	Patra et al. (2016) [21]
Manure management	CH_4_	(0.19–0.37) kg/head/year	kg CH_4_/year	IPCC (2006)
Manure management direct	N_2_O	1.1 kg N_2_O-N/kg N Deep litter	kg N_2_O/year	IPCC (2006)
Manure management—indirect	N_2_O	kg N_2_O-N/volatilized 0.0075 kg N_2_O-N/leaching	kg N_2_O/year	IPCC (2006)
N from inorganic fertilization	N_2_O	0.001 kg N_2_O-N (kg N input)	kg N_2_O/year	IPCC (2006)
N from organic manure)	N_2_O	0.001 kg N_2_O-N (kg N input)	kg N_2_O/year	IPCC (2006)
N from urine and dung inputs to grazed soils	N_2_O	0.001 kg N_2_O-N (kg N input)	kg N_2_O/year	IPCC (2006)
Indirect emissions management soils	N_2_O	0.001 kg N_2_O-N (kg N input)	kg N_2_O/year	IPCC (2006)

IPCC; The Intergovernmental Panel on Climate Change, CH_4;_ methane, N_2_O; nitrous oxide.

### Statistical analysis

Data were statistically analyzed using analysis of variance (ANOVA) in XLSTAT software for testing the significant variation among three grazing system (intensive, semi-intensive and extensive systems) at probability level of 0.05. Least Significant Difference (LSD) was conducted if significant effects of treatment set at an alpha level of 0.05 were found.

## Results and discussion

### Input variation in grazing systems and GHG emissions

The grazing systems varied largely w.r.t. fodder intake, concentrate and grazing hours per day, in intensive (IS) and semi-intensive (SIS) system, the intake of concentrate was higher in IS, whereas, total fodder intake hours was higher in extensive system (ES) by approximately 54.7% and 27.5% over IS and SIS, respectively due to more grazing (Table 1).

The gain in body weight was lowest in ES due to no concentrate supplementation, poor quality fodder intake and low digestibility of the feed resources. Among all inputs, concentrate feed resulted in highest CFs however, reducing the use of concentrates resulted in reduce growth rates which affected overall farm productivity and at the same time increased dependency on pastures thereby leading to increased GHG emissions from the extensive system [7]. Though fodder intake was high in ES but the total dry matter intake was higher in SIS (21.4%) followed by IS (6.1%). Higher dry matter intake resulted in higher average daily gain of sheep in SIS which was around 15.6 and 126.5% higher as compared to IS and ES, respectively (Table 1). EntericCH_4_production is mainly governed by fodder intake, diet composition and digestibility [8]. CH_4_ emissions when accounted as per sheep per day, per kg dry matter intake and per kg digestible dry matter intake was significantly higher in extensive system due to increased roughage intake with high fibre content, poor digestibility and longer retention time of feed in the rumen [9] which ultimately resulted in higher CH_4_ production. The extensive system was based on grazing on natural pastures so their input requirement was very low, the consumption of electricity was almost 14-fold lower over IS and fertilizer consumption was nil (Table 4). Vagnoni et al. [10] also reported that field operations, electricity and machineries affected carbon foot prints of Sardinian dairy sheep production systems at different input levels.

**Table 4:** Breakdown of emission source of carbon foot prints (kg CO_2_-eq) of different grazing system followed in semi-arid region of India.

Emission sources breakdown	Intensive system	Semi-intensive system	Extensive system
**CO**_**2**_ **form manufacture of inputs**			
Fuel	0.80	0.39	0.33
Electricity	0.81	0.70	0.23
Bedding materials	0.04	0.02	0.01
**Mixed GHGs from growth of inputs**			
Concentrates and other feeds	0.66	0.24	0.00
**Inputs total**	**2.31**	**1.34**	**0.58**
**N**_**2**_ **O emission from soils**			
**Direct emission**			
Excreta	1.77	1.69	1.84
Feed residues	3.70	3.83	3.61
**Indirect emission**			
Volatilization loss	0.48	0.42	0.46
Leaching and run off	0.019	0.03	0.016
**N**_**2**_ **O emission from manure storage**			
Direct	0.23	0.22	0.24
Indirect	0.035	0.03	0.036
**N**_**2**_ **O total**	**6.23**	**6.22**	**6.20**
**CH**_**4**_ **emission**			
Enteric fermentation (kg/animal/yr)	7.39	8.47	9.34
Excreta	0.94	0.91	0.98
**CH**_**4**_ **Total**	8.33	8.30	10.31
**Total Carbon footprints**	16.88	15.87	17.09

CH_4;_ methane, N_2_O; nitrous oxide, CO_2_; carbon dioxide

### Carbon foot prints influenced by different grazing systems

The total C foot print (kg CO_2_-eq) was estimated to be 16.9, 15.8 and 17.1 in intensive, semi-intensive and extensive system of grazing, respectively (Table 4). The SIS was reported to be most C efficient system as it recorded 5.3 and 9.4% of total CFs as compared to IS and ES, respectively. Enteric fermentation was the chief contributor to CFs irrespective of systems, it was around 46.4, 56.1 and 56.5% of total CFs recorded in IS, SIS and ES, respectively. In previous studies it was reported that, in sheep 61 to 68% GHG emission is from enteric CH_4_ production which mainly depends on feed digestibility and efficiency of rumen fermentation [11]. According to Eckard et al. [12] and Martin et al. [13] feeding of grain-based diets to ruminants having higher starch content, reduces CH_4_ production in two ways, firstly, it enhances intake of feed and improves digestibility and secondly, supports the production of propionate in rumen, which acts as an alternate hydrogen sink and reduces methanogenesis. Benchaar et al. [14] estimated CH_4_ production in ruminants can be reduced upto 1–3% by increasing the proportion of concentrate in diets. However, the applicability of high-concentrates diets is restricted to IS, therefore, SIS may be a viable option for semi-arid Indian conditions.

**Fig 2:**
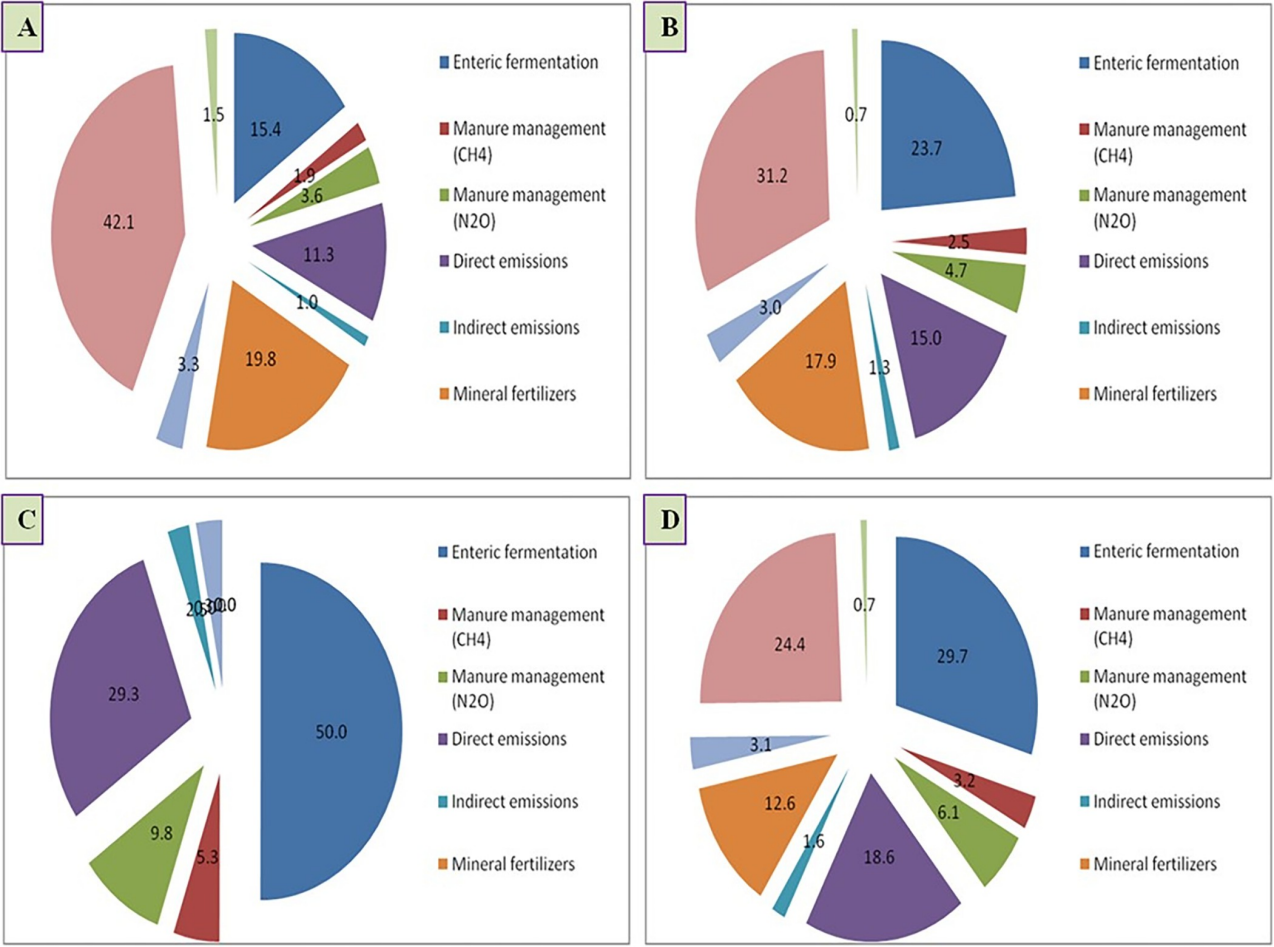


The second largest contributor was direct N_2_O emission from soils; it resulted in 17.3% of total CFs, irrespective of the grazing system (Fig 2). Further, among direct N_2_O emission, feed residues contributed more than excreta (Table 4). According to Schils et al. [15] nitrogen enters into soil through animal excreta, manure fertilizer application and crop residues, N losses can occur directly as volatilization and denitrification or indirectly through leaching and runoff. In Scotland, it was estimated that 0·017 N from applied fertilizer and excreta was emitted as N_2_O [16]. After CH_4_ and N_2_O emission, CF from input contribution was least and the values were almost negligible in extensive system. The contribution of inputs varied from 14.5% in IS, 8.9% in SIS and 0.03% in ES, respectively. The input contribution in ES was lowest because of no concentrate supplementation and less use of fuel and electricity.

It was evident from Table 5 that in IS, purchased feed contributed maximum (42%) towards CO_2_ eq for 1 kg of mutton production, whereas, it was maximum for enteric fermentation (50%) in ES. In SIS, when animals were stall fed with grazing, the share of purchased feed was reduced (31%). On an average, 30% of emission was from enteric fermentation followed by purchased feed (24%) in SIS. Manure management resulted in 3 and 6% CH_4_ and N_2_O emission regardless of system (Fig 2). Electricity contributed around 3% and other inputs share only 0.7% contribution. In previous reports, it was documented that for 1 kg mutton production, the CF 26 to 33 kg CO_2_-eq in Tunisia [17], 39–52 kg CO_2_-eq in Spain [18] and global average was estimated to be 24 kg CO_2_-eq [1] with the maximum contribution from CH_4_ and N_2_O due to enteric fermentation and manure management [19].

**Table 5:** Carbon foot prints from different inputs given both as kg CO2 kg-1 mutton and % of total carbon foot prints in parenthesis.

Source of emission (kg CO_2_eq kg^-1^mutton)	Intensive system	Semi-intensive system	Extensive system
Enteric fermentation (CH_4_)	0.38 (15)	0.41(22)	0.58(50)
Manure management (CH_4_)	0.047(2)	0.044(2)	0.061(5)
Manure management (N_2_O)	0.090(4)	0.081(4)	0.114(10)
Direct emissions (N_2_O)	0.28(11)	0.26(14)	0.34(29)
Indirect emissions(N_2_O)	0.025(1)	0.022(1)	0.029(3)
Mineral fertilizers	0.49(20)	0.31(16)	0.00(0)
Energy (Oil + Electricity)	0.081(3)	0.052(3)	0.035(3)
Feed purchased	1.04(42)	0.69(37)	0.00(0)
Other inputs	0.036(1)	0.012(1)	0.00(0)
**Total emissions**	2.469	1.881	1.159

CH_4;_ methane, N_2_O; nitrous oxide

## Conclusions

Our study demonstrated the importance of CFs estimation by LCA under different sheep farming system of semi-arid regions in India. The total CFs were estimated to be 16.9, 15.8 and 17.1 kg CO_2_-eq in intensive, semi-intensive and extensive system of grazing indicating semi-intensive system to be most carbon (C) efficient. Results suggested that adoption of improved grazing management i.e. semi-intensive system for mutton production system has lowest carbon cost. However, development of strategies for management and improvement of pastures should be given importance, especially in areas where livestock is the backbone to small and marginal farmers. Extensive system followed for sheep rearing should be improved by pasture management strategies or farmers should shift to semi-intensive system for quality production, sustainability and sound environment.
